# Clinical and sociodemographic factors related to the timing of autism diagnosis in an Italian cohort of children

**DOI:** 10.3389/fpsyt.2025.1638341

**Published:** 2025-09-04

**Authors:** Alessia Cianfa, Silvia Guerrera, Elisa Fucà, Emma Annechini, Valentina Napoli, Stefano Vicari, Giovanni Valeri

**Affiliations:** ^1^ Child Adolescent Neuropsychiatry Unit, Bambino Gesù Children’s Hospital IRCCS, Rome, Italy; ^2^ Child Neurology and Psychiatry Unit, Fondazione Policlinico Universitario Agostino Gemelli IRCCS, Rome, Italy; ^3^ Life Sciences and Public Health Department, Catholic University, Rome, Italy

**Keywords:** early identification, diagnosis timing, clinical predictors, parental recognition, sociodemographic factors, early intervention

## Abstract

**Introduction:**

The onset of features associated with “Autism Spectrum Condition” can vary significantly in both timing and presentation. A formal diagnosis often does not align with the emergence of early signs due to challenges in recognizing the initial manifestations of neurodevelopmental differences. Current research shows limited consensus regarding clinical and sociodemographic factors linked to early versus late diagnoses, underscoring the need for further investigation.

**Methods:**

Data were retrospectively collected from clinical records of children evaluated for suspected autism at the Child and Adolescent Neuropsychiatry Unit of an Italian pediatric hospital between 2016 and 2023. The standardized evaluation included neuropsychiatric examination, assessment of cognitive and adaptive functioning, evaluation of autistic traits, and a comprehensive psychopathological profile. Correlational analyses examined clinical and sociodemographic variables associated with diagnosis timing, while a linear regression model was used to identify independent predictors. Inclusion criteria included a first diagnosis of autism or high likelihood of autism, and an age between 18 and 71 months. Exclusion criteria included known genetic conditions or a prior autism diagnosis.

**Results:**

The final sample included 150 children (mean age: 43.71 ± 13.6 months; 123 males, 27 females). Among clinical variables, cognitive and developmental differences and parental recognition of early communication variations were linked to earlier diagnosis, while a distinct emotional-behavioral profile was associated with later diagnosis. Regarding sociodemographic factors, being a first-born child and higher parental stress were correlated with delayed diagnosis. Parental age and education showed no significant associations. Only cognitive and developmental profiles, along with early symptom recognition (ADI-D), emerged as the strongest predictors of early diagnosis. Conclusions: These results emphasize the critical need to enhance early identification of autism and to minimize the adverse effects associated with delayed diagnosis. They also underline the clinical relevance of caregiver education—particularly for first-time parents—as a strategy to facilitate timely recognition and intervention.

## 1 Introduction

Autism is a lifelong neurodevelopmental condition characterized by challenges in social communication and the presence of restricted, repetitive behaviors or interests, with traits typically becoming apparent before the age of three (1).

The “Autism and Developmental Disabilities Monitoring” network of the Centers for Disease Control and Prevention (CDC) suggests that approximately 1 in 31 children are diagnosed with autism, reflecting a substantial increase from the prevalence of 1 in 150 reported in 2000 (2). A systematic review of studies on the global prevalence of autism highlights that estimates are influenced by a complex interplay of sociodemographic factors, including sex, socioeconomic status, geography, ethnicity, and nativity (3). These variations are shaped by differences in community awareness, healthcare capacity, and access to services, underscoring the importance of addressing these factors to improve healthcare policies and reduce disparities in autism diagnosis and treatment (3).

Autism manifests significant clinical heterogeneity influenced by multiple factors, including intellectual and linguistic functioning, as well as co-occurring medical or psychiatric conditions (4–6). This variability reflects the complex interplay of genetic, epigenetic, and environmental influences (7–9) and poses important challenges for early recognition and diagnosis. A combination of core characteristics and co-occurring conditions can shape the overall level of support needs experienced by autistic individuals, with implications for both the individuals themselves and society at large (1, 7, 8). Early signs of autism often emerge by 12 months of age; however a formal diagnosis is typically made between 18 and 24 months, with diagnostic outcomes remaining stable over time; nevertheless, some individuals may receive a diagnosis later in life, particularly when increasing social demands exceed their adaptive capacities, making the behavioral manifestations of the condition more apparent (4, 5, 9).

It is important to acknowledge that many autistic individuals—especially females or those with higher adaptive skills— may receive a diagnosis later in life and are thus likely underrepresented in preschool-aged samples examined in the literature (10–13). This represents a key limitation in interpreting age-at-diagnosis findings.

Early diagnosis is crucial, given that extensive evidence demonstrates that timely intervention can significantly enhance developmental outcomes (6, 14). Notably, an autism diagnosis before 3 years of age is associated with marked improvements, particularly in social functioning, potentially due to heightened neuroplasticity and behavioral adaptability observed in younger children, which may enable them to derive greater benefit from early intervention efforts (5, 15). Nonetheless, the early diagnosis of this condition remains a considerable challenge, with estimates indicating that delays in diagnosis are still prevalent (16). A systematic review and meta-analysis of 35 studies from 2012 to 2019 indicated a global average age at diagnosis of 60.48 months, despite guidelines advocating for earlier intervention (16).

A substantial body of literature indicates that a combination of clinical and sociodemographic factors significantly influences the age at which autism is diagnosed (9, 16, 17). The level of expression of autistic traits may play a crucial role in the timing of diagnosis. Indeed, children exhibiting more pronounced traits, such as marked difficulties in social communication and the presence of repetitive behaviors, generally receive earlier diagnoses compared to those with less evident traits (9, 16, 18).

Delays in language acquisition are often a major concern for parents and frequently serve as a primary reason for seeking diagnostic evaluation, thereby facilitating access to diagnostic services (9, 19).

Developmental regression, characterized by the loss of previously acquired skills, particularly in social or language domains, is another critical factor contributing to the early identification of autism (9, 19).

The presence of comorbidities is also recognized as a variable associated with the timing of diagnosis. However, literature presents conflicting results: some studies suggest that comorbidities conditions, such as global developmental delay or intellectual disabilities (10, 11, 20), as well as language delay or regression (12, 19, 20) are associated with earlier diagnoses. Conversely, other research highlights that children diagnosed with autism at a later stage exhibit a profile of global developmental delay which can hinder the identification of the core features of the condition (19–22). These children often have a history of language difficulties or neurodevelopmental conditions such as Attention Deficit Hyperactivity Disorder (ADHD), which may overlap with or contribute to the presentation of autistic traits (16, 22–25).

Gender has also been identified as a variable associated with the timing of autism diagnosis. Several studies indicate that females typically receive the diagnosis at a later age compared to males (9, 16, 17, 19, 26). However, findings regarding this variable are not consistent within the scientific literature (9, 19).

The age at diagnosis is also significantly influenced by the timing of parental concerns, with earlier parental concerns associated with quicker diagnoses (9, 19, 27). Additionally, birth order plays a role, as studies suggest that firstborn children may receive diagnoses at a later age compared to their younger siblings (9).

Furthermore, an increasing body of research highlights the potential role of sociodemographic factors and cultural barriers in influencing diagnostic timing (3, 7, 9, 19). Delays in obtaining a diagnosis contribute significantly to elevated stress levels among parents of children with autism, who often experience higher stress compared to parents of typically developing children or those with other chronic conditions (28, 29).

Overall, the reviewed literature does not identify a specific profile associated with early or late autism diagnosis. Instead, it suggests that a complex interplay of clinical and environmental variables may be crucial in determining diagnostic timing.

These factors can contribute to delays in both identification and intervention, emphasizing the need for further investigation into the clinical and sociodemographic determinants of early versus late autism diagnosis. In this study, we investigated clinical and sociodemographic factors associated with and predictive of early versus late autism diagnosis within an Italian cohort of autistic children. We identified two main objectives:

a) To assess the relationship between age at first diagnosis and clinical features such as the level of expression of autistic traits, cognitive functioning, adaptive skills, and emotional and behavioral challenges, as well as gender;
b) To investigate the relationship between age at first diagnosis and sociodemographic factors: maternal and paternal age, maternal and paternal education, number of siblings, birth order, and maternal stress.

Understanding these factors is crucial for improving diagnostic practices and promoting timely interventions that can enhance the prognosis for children with autism.

## 2 Materials and methods

### 2.1 Procedure

Data were retrospectively collected from an in-depth review of the files of patients who referred to the Child and Adolescent Neuropsychiatry Unit of a third level Children’s Hospital between 2016 and 2023 for a neuropsychiatric evaluation following pediatrician’s clinical suspicion of autism.

Speech and language delay is often the primary concern prompting referral in suspected cases of autism condition. For instance (13), report that speech delay was the main reason for initial medical consultation in the vast majority of cases, typically occurring alongside early social and communicative impairments.

Routine assessment procedure always included neuropsychiatric examination, cognitive and adaptive functioning evaluation, assessment of autistic traits and an accurate psychopathological investigation. Questionnaire completion was part of the standard clinical assessment protocol routinely conducted at our Neuropsychiatry Unit. All caregivers provided written informed consent prior to the assessment, in accordance with institutional protocols and international ethical guidelines for clinical research. To ensure data quality, the plausibility and completeness of questionnaire responses were reviewed by the clinical team during the assessment process. In cases of missing or ambiguous responses, clarification was sought directly from the caregivers during clinical interviews. This procedure helped minimize data gaps and ensured the reliability of the information included in the study.

The inclusion criteria were as follows: individuals who were receiving a diagnosis of autism for the first time, based on gold-standard diagnostic tools, or those who were identified as having a likelihood of receiving an autism diagnosis, with subsequent confirmation of the diagnosis; age between 18 and 71 months. Exclusion criteria were as follows: presence of genetic conditions; the presence of a previously established diagnosis of autism.

The study was conducted according to the guidelines of the Declaration of Helsinki and approved by the local Ethics Committee (protocol code: 2423_OPBG_2021, approved on 27 October 2021).

### 2.2 Participants

The final sample included 150 children referring to our Neuropsychiatry Unit who received a first diagnosis of autism (mean age: 43.71 ± 13.6 months; 123 males, 27 females). Fifty-eight participants (39% of the total sample) received the diagnosis before 36 months of age (mean IQ/DQ = 65.51 ± 15.5; mean ADOS - 2 Calibrated Severity Score for Social Affect = 6.6 ± 1.5; mean ADOS - 2 Calibrated Severity Score for Restricted and Repetitive Behaviors = 6.3 ± 1.5; mean ADI-R scores domain A = 13.4 ± 3.9; mean ADI-R scores domain B = 9 ± 3.1; mean ADI-R scores domain C = 4.2 ± 1.7; mean ADI-R scores domain D = 4.7 ± 0.58). Ninety-two participants (61%) were diagnosed after 36 months of age (mean IQ/DQ = 65.81 ± 27; mean ADOS - 2 Calibrated Severity Score for Social Affect = 6.2 ± 1.3; mean ADOS - 2 Calibrated Severity Score for Restricted and Repetitive Behaviors = 6.8 ± 1.3; mean ADI-R scores domain A = 13.6 ± 4.4; mean ADI-R scores domain B = 9.5 ± 2.7; mean ADI-R scores domain C = 4.6 ± 1.8; mean ADI-R scores domain D = 4.3 ± 0.84).

Demographic features of parents are summarized in **Table 1**.

**Table 1:** Demographic features of mothers and fathers of the participants.

Demographic Variables	Mothers	Fathers	p
Age	37.53 ± 5.89	40.62 ± 6.63	<0.01
Education (in years)	14.07 ± 3.45	12.87 ± 3.47	0.003
Employment (%)	Not employed = 38.62	Not employed = 0.67	0.012
	Lower supervisory, technical, (semi) routine, others = 8.97	Lower supervisory, technical, (semi) routine, others = 10.07	
	Intermediate, small employers, own accountants = 34.48	Intermediate, small employers, own accountants = 47.65	
	Managerial/professional = 17.93	Managerial/professional = 41.61	

### 2.3 Measures

#### 2.3.1 Autistic symptoms assessment

The diagnosis of autism was established in accordance with the DSM - 5 and was confirmed by the administration of the “gold-standard” instruments for the assessment of autistic traits, namely the Autism Diagnostic Observation Schedule, Second Edition (ADOS - 2) (30) and the Autism Diagnostic Interview-Revised (ADI-R) (31). Systematic reviews and meta-analysis confirm their high validity and reliability in clinical diagnostic contexts (32).

The ADOS - 2 is a semi-structured direct assessment of communication, social interaction, and play or imaginative use of materials for individuals with a suspected diagnosis of autism. The ADOS - 2 consists of five modules designed for children and adults with different levels of language, from nonverbal to verbally fluent; it was administered and scored by licensed clinicians. The total score is derived from the “Social Affect” and “Restricted and Repetitive Behaviours” domains. In the analyses, the Calibrated Severity Scores (CSS) were considered for the ADOS - 2.

The ADI-R is a standardized, semi‐structured interview during which caregivers report information about an individual suspected to be on the autism spectrum. The instrument generates algorithm scores for each of the three subdomains of autistic traits: qualitative challenges in reciprocal social behavior (A); qualitative abnormalities in communication (B) and restricted range of interests and/or stereotypic behaviors (C). Additionally, there is a scale that indicates “developmental anomalies evident at or before 36 months” (D). Data from ADI-R were available for a subgroup of 136 participants.

Due to the retrospective nature of the study, ADI-R data were only partially available, as the instrument was not consistently used across all clinical records. This constitutes a methodological limitation. Despite this, we believe that the analyses remain robust, as the available data were carefully examined, and other standardized measures were utilized to complement the assessment.

#### 2.3.2 Cognitive assessment

Cognitive development was assessed by Griffiths Scales of Child Development, Third Edition (33). Griffiths III provides an overall measure of a child’s development, as well as an individual profile of strengths and needs across five areas: Foundations of Learning – assesses critical aspects of learning during the early childhood years; Language and Communication – measures overall language development, including expressive language, receptive language and use of language to communicate socially with others; Eye and Hand Coordination – considers fine motor skills, manual dexterity and visual perception skills; Personal–Social–Emotional – measures constructs relating to the child’s developing sense of self and growing independence, interactions with others, plus many aspects of emotional development; Gross Motor – assesses postural control, balance and gross body coordination, among other abilities.

For children over the age of 3, cognitive level was assessed through the Leiter International Performance Scale – 3rd Edition - Leiter-3 (34) – which provides a nonverbal measure of intelligence and assesses the ability to reason by analogy, by matching and perceptual reasoning in general, irrespective of language and formal schooling. The Global Non-Verbal Intelligent Quotient obtained through this test is based on four subtests: Figure Ground, Form Completion, Classification and Analogies, and Sequential Order.

#### 2.3.3 Psychopathological and behavioral screening

Emotional and behavioral problems were evaluated by means of the Child Behavior Checklist (CBCL) (35, 36). The CBCL for ages 1.5 to 5 consists of 100 problem items. The instrument generates seven syndrome scales and five DSM-oriented scale profiles, consistent with the diagnostic categories of DSM-IV-TR and DSM - 5. In the current study, we considered the seven syndrome scales, namely: (1) emotionally reactive; (2) anxious/depressed; (3) somatic complaints; (4) withdrawn; (5) sleep problems (CBCL only); (6) attention problems; and (7) aggressive behavior. In addition, there are five DSM-oriented scales: (1) affective problems; (2) anxiety problems; (3) pervasive developmental problems; (4) attention-deficit/hyperactivity problems; and (5) oppositional defiant problems.

The CBCL is widely used to assess emotional and behavioral problems in children with Autism. Previous studies has documented a high prevalence of such difficulties in this population, confirming the CBCL’s utility as a clinical screening tool (37).

#### 2.3.4 Adaptive functioning

The Adaptive Behavior Assessment System – Second Edition (ABAS-II) (38) was used to investigate adaptive skills. This parent/caregiver report questionnaire consists of eleven skill areas organized into three general domains: conceptual, practical, and social. The composite and domain scores are standard scores with a norm-referenced mean of 100 and standard deviation of 15.

Assessment of adaptive skills is crucial in Autism conditions, as these abilities are often impaired in this population. Recent studies have used the ABAS-II to identify significant adaptive behavior deficits in children with Autism, highlighting the importance of combining this assessment with cognitive evaluations to achieve a comprehensive diagnostic profile (39).

#### 2.3.5 Maternal stress assessment

To investigate maternal stress levels, the Parenting Stress Index-Short Form (PSI-SF) (40) was used. PSI is an easy-to administer tool to measure maternal stress. It consists of 36 questions and each item is rated on a 5-point Likert scale from (1) strongly disagree to (8) strongly agree. The PSI captures three domains—parental distress (PD), parent–child dysfunctional interaction (P-CDI), and difficult child (DC). The sum of all questions results in the Total Stress score.

The PSI-SF was selected as a validated and widely used instrument for assessing parental stress. It has been translated into several languages and is frequently employed in autism research (41). Its use is particularly well supported in the context of autism spectrum conditions, with numerous studies confirming its appropriateness for measuring stress in parents of children on the autism spectrum (42, 43).

### 2.4 Statistical analyses

Descriptive statistics were used to analyze demographic and clinical characteristics of the whole sample. Chi-squared and t-test was used to investigate group differences. Pearson and Spearman correlation analyses were used to explore the association between the age at first diagnosis and child’s individual and clinical features as well as between the age at first diagnosis and parental and family features. Group differences were examined by t test. A linear regression analysis examined a model of the variables predicting the age of the autism diagnosis. More in detail, the independent variables of the regression analysis were chosen according to the results of the correlation analyses. Therefore, IQ/DQ, social domain composite score of the ABAS II, ADI-A, ADI-D, birth order, and some CBCL scales (Emotionally reactive, Anxious/depressed, Somatic complaints, Attention problems, Aggressive behavior) were entered as the independent variables, whereas the age of autism diagnosis was entered as the dependent variable. A p-value less than or equal to 0.05 was considered as statistically significant.

## 3 Results

### 3.1 Association between age at first diagnosis and child’s individual and clinical features

In order to determine the relationship between age at first diagnosis and selected child individual features (i.e. IQ, adaptive level, emotional and behavioral problems, autistic traits) we performed Spearman correlations.

We found a significant positive correlation with IQ (r = 0.177; p = 0.03) and negative correlation with social domain composite score of the ABAS II (r = -0.261; p = 0.001).

No associations emerged between age at first diagnosis and the other domains of the ABAS II (all p > 0.05).

The analysis of the association between age at first diagnosis and ADOS - 2 CSS failed to detect significant association with Social Affect (r = -0.099; p = 0.39) and Restricted and Repetitive Behaviors (r = 0.116; p = 0.315) domains.

On the other hand, we found significant association between the age at first diagnosis and some ADI-R scores.

The results are summarized in **Table 2**. We also found significant associations between age at first diagnosis and parent-reported emotional and behavioral problems in several areas. The results are summarized in **Table 3**.

**Table 2:** Association between age at first diagnosis and ADI-R scores.

	ADI-A (r, p)	ADI-B (r, p)	ADI-C (r, p)	ADI-D (r, p)
Age at first diagnosis	0.05, 0.56	0.191, 0.026*	0.133, 0.122	-0.283, 0.001**

* p < 0.05; ** p < 0.01. ADI-A, Autism Diagnostic Interview-Revised – area of reciprocal social behavior; ADI-B, Autism Diagnostic Interview-Revised – area of communication; ADI-C, Autism Diagnostic Interview-Revised – area of restricted range of interests and/or stereotypic behaviors; ADI-D, Autism Diagnostic Interview-Revised – area of early history.

**Table 3:** Association between age at first diagnosis and parent-reported emotional and behavioral difficulties (CBCL scores).

	Emotionally reactive (r, p)	Anxious/ depressed (r, p)	Somatic complaints (r, p)	Withdrawn (r, p)	Sleep problems (r, p)	Attention problems (r, p)	Aggressive behavior (r, p)
Age at first diagnosis	0.286, <0.001**	0.327, <0.001**	0.173, 0.034*	0.047, 0.571	0.001, 0.99	0.169, 0.04*	0.255, 0.002**

* p < 0.05; ** p < 0.01.

No differences between males and females emerged in the age of first diagnosis (44.5 ± 13.69 and 40.1 ± 12.81, respectively; p = 0.118). Of note, there was an unequal sex representation in our sample (123 males vs 27 females).

### 3.2 Association between age at first diagnosis and parental and family features

In order to determine the relationship between age at first diagnosis and selected parental and family features (i.e. maternal age, paternal age, maternal education, paternal education, number of siblings, birth order, maternal stress) we performed Pearson correlations and Spearman correlations, when required.

We did not find association with maternal age (r = 0.068; p = 0.407) nor paternal age (r = 0.058; p = 0.478). No associations emerged between age at first diagnosis and maternal education (r = -0.060; p = 0.465) nor paternal education (r = 0.049; p = 0.549), nor number of siblings (r = 0.038; p = 0.654).

A significant negative association with birth order emerged (r = -0.203; p = 0.013). **Table 4** summarizes the correlations between age at first diagnosis and maternal stress.

**Table 4:** Association between age at first diagnosis and maternal stress (PSI scores).

	PD (r, p)	P-CDI (r, p)	DC (r, p)	Total score (r, p)
Age at first diagnosis	0.235, 0.004**	-0.013, 0.875	0.265, 0.001*	0.235, 0.004**

* p < 0.05; ** p < 0.01. PD, parental distress; P-CDI, parent–child dysfunctional interaction; DC, difficult child.

### 3.3 Predictors of age at first diagnosis

The above analyses indicated significant associations between age at first diagnosis of autism and multiple variables, namely child’s IQ/DQ, social domain composite score of the ABAS II, ADI-B, ADI-D, birth order, and some CBCL scales (Emotionally reactive, Anxious/depressed, Somatic complaints, Attention problems, Aggressive behavior). Therefore, we aimed to determine if they could be considered predictors of the age of diagnosis. To address this issue, a linear regression analysis was performed. The results are summarized in **Table 5**. Linear regression analysis revealed that some features significantly predicted the age at first autism diagnosis, accounting for the 22% of variance (adjusted r-square; p < 0.01), with only ADI-D and child’s IQ/DQ having significant predictive values (see **Table 5**).

**Table 5:** Regression analysis for the prediction of age at first diagnosis.

		AGE AT DIAGNOSIS		
Model	Variables	B	SE	p
1	IQ/DQ	0.202	0.045	0.018
	Social domain composite score (ABAS-II)	-0.164	0.074	0.07
	ADI-B	0.12	0.373	0.161
	ADI-D	-0.265	1.397	0.002
	Emotionally reactive	0.117	0.202	0.4
	Anxious/depressed	0.084	0.258	0.479
	Somatic complaints	0.039	0.175	0.688
	Attention problems	-0.018	0.144	0.855
	Aggressive behavior	0.105	0.238	0.357
	Birth order	-0.098	0.65	0.218

IQ/DQ, Intelligence Quotient/Developmental Quotient; ABAS-II, Adaptive Behavior Assessment System – Second Edition; ADI-B, Autism Diagnostic Interview-Revised – area of communication; ADI-D, Autism Diagnostic Interview-Revised – area of early history.

## 4 Discussion

This study aimed to explore the factors influencing the timing of autism diagnosis, with a focus on clinical and sociodemographic variables in a cohort of 150 Italian autistic preschoolers.

### 4.1 Clinical variables and age at first diagnosis

The first finding of this study indicates a significant positive correlation between children’s cognitive level and the age at which autism was diagnosed. Specifically, children with a lower IQ/DQ were diagnosed at an earlier age. This finding suggests that global developmental delays may enhance the expression of the overall clinical manifestations, facilitating earlier recognition by families and healthcare professionals. This observation is consistent with a substantial body of existing literature emphasizing the pivotal role of IQ in the diagnostic processes for autism (10, 25, 44).

A large-scale study conducted by Denisova and Lin (10), involving 8,000 children aged between 2 and 68 months, confirmed that cognitive abilities, measured in terms of DQ, were consistently lower in children with autism compared to typically developing (TD) children, and these scores were significantly correlated with the presence of both social and non-social features of autism.

Moreover, evidence from studies such as that of Saban-Bezalel et al. (25) has demonstrated that lower DQ and IQ scores, particularly those below 70, are associated with earlier diagnosis. Conversely, a separate body of literature suggests that global developmental delay or low IQ may contribute to a delayed diagnosis, often due to the masking of core autism symptoms by other conditions (19–22), and that children diagnosed later tend to exhibit poorer overall cognitive abilities at school age, compared to those diagnosed earlier (45).

A study conducted by Miller et al. (21), which compared the development and autistic traits in very young children diagnosed at different ages—early (12 – 18 months), middle (19 – 24 months), and later (25 – 41 months)—supports the finding that children diagnosed later tend to exhibit more pronounced cognitive challenges compared to those diagnosed earlier.

Variations in findings across studies may be due to differences in sample characteristics and assessment tools. For instance, Miller et al. used the “Mullen Scales of Early Learning” and focused on age groups with a lower average age than in our study. Including very young children — for whom developmental gaps may not yet be fully apparent — and comparing groups across different age ranges may have enhanced the visibility of such differences. The authors interpret their results through Landa et al.’s model of “progressive divergence from typical development,” which suggests that developmental challenges emerge and intensify over time (44).

These findings may reflect both the natural course of developmental difficulties and the cumulative effects of delayed diagnosis and postponed intervention. Considering the likelihood of challenging developmental trajectories, the results of the present study suggest that more pronounced cognitive difficulties may actually facilitate earlier diagnosis, highlighting significant implications. On one hand, this suggests that more pronounced challenges may lead to a timelier initiation of therapeutic interventions, potentially yielding positive effects on developmental outcomes. On the other hand, it underscores the likelihood of underdiagnosis in children with autism who do not exhibit significant cognitive delays, emphasizing the need for more sensitive diagnostic approaches capable of identifying the full spectrum of autism manifestations.

We also observed a significant negative correlation between the timing of autism diagnosis and the composite score related to the social domain of the ABAS II adaptive functioning test. Within our sample, poorer social adaptive functioning was associated with a later diagnosis. To the best of our knowledge, this is the first study investigating the potential correlation between adaptive functioning and the age of autism diagnosis.

Numerous studies have shown that adaptive functioning is significantly impaired in autism (46–48), particularly in the area of social skills (49, 50), and these impairments tend to occur more frequently than in other neurodevelopmental conditions (50).

Our findings are consistent with those reported by Miller et al. (21), who observed that the group with a later diagnosis showed poorer adaptive skills. This convergence of evidence suggests that the age at which a diagnosis is made may have a significant impact on the trajectory of adaptive functioning. Specifically, children diagnosed at a later age may experience greater difficulties in the social domain, likely due to the accumulation of missed developmental opportunities associated with core features as well as the absence of interventions during critical developmental windows. This trend is supported by a recent longitudinal Italian study by Casula et al. (48) involving a large sample of preschool-aged children with autism, which found a deterioration in adaptive functioning, especially in the social and practical domains, associated with an increase in autistic traits.

These findings underscore the importance of considering not only how late diagnosis affects core autistic traits but also how it influences broader domains of functioning such as social adaptation. In our study, no significant correlation was found between the level of expression of autistic traits, as measured by the ADOS-2, and the timing of diagnosis.

This finding is inconsistent with the majority of studies in the literature, which indicate that the level of expression of autistic traits correlates with the age at diagnosis, showing that earlier diagnoses are associated with a higher level of core traits (9, 16, 18, 22, 51), in particular regarding the area of social communication (9).

The discrepancy between our finding and those reported in the previously mentioned literature may be partially explained by the clinical characteristics of our sample. Given that our sample consisted of preschool-aged children, it is plausible—consistent with prior research (9, 16, 18)—that the core features of autism were more pronounced in this group compared to children diagnosed at school age or later, in whom the expression of autistic traits may be less noticeable. This may have led to reduced variability, potentially limiting the ability to detect significant differences in relation to the timing of diagnosis.

Additionally, the clinical uniformity of the sample—ensured by prior pre-screening conducted by child neuropsychiatrists—may have further reduced interindividual differences, potentially obscuring associations between ADOS-2 scores and the timing of diagnosis. A recent meta-analysis (32) confirms that the ADOS-2, demonstrates high validity and sensitivity, particularly in research settings, where sensitivity ranges from 0.89 to 0.92 and specificity from 0.81 to 0.85. Compared to the ADI-R, the ADOS-2 generally shows superior diagnostic performance. However, its accuracy appears more variable in clinical settings, revealing inconsistencies outside of controlled research environments. While underscoring the strong diagnostic utility of the ADOS-2, the meta-analysis also highlights the need for further studies assessing its effectiveness in routine clinical practice, where diagnostic challenges may differ significantly.

The absence of a correlation between symptom severity (as measured by the ADOS-2) and the timing of diagnosis may also reflect emerging evidence that certain clinical signs—though not core features of autism—serve as more visible early warning signals. These signs often prompt earlier professional attention and facilitate timely identification. The study by Sicherman et al. (52), based on a large cohort, found that early communication difficulties—such as poor response to name, lack of gestures, and delayed speech—were among the strongest predictors of earlier diagnosis, often occurring before age two. Notably, other non-specific developmental signs—including motor delays, sleep disturbances, and sensory sensitivities—also played a critical role in prompting clinical evaluations, particularly when observed by experienced professionals. In contrast, behaviors such as aggression, severe tantrums, and insistence on sameness tend to emerge later in more socially complex contexts and were associated with delayed diagnoses. These findings support a broader approach to developmental monitoring, in which indirect or non-core signs are recognized as clinically meaningful cues for early autism detection.

In line with this, a recent study by Hrdlicka et al. (53), conducted with children aged 2 to 16 years, also found that earlier diagnoses were associated with higher ADOS-2 social domain scores, increased restricted and repetitive behaviors, and certain sociodemographic factors—such as higher maternal education and parental cohabitation. Their analysis explored how such variables might influence the relationship between symptom severity and timing of diagnosis. Moreover, the wide age range in their sample may have introduced greater variability in symptom presentation at the time of initial evaluation.

Altogether, these considerations underscore the multifactorial and complex nature of assessing the severity of clinical manifestations in autism. This complexity likely explains the lack of a direct association between core symptom severity and timing of diagnosis, which is shaped by a wide array of interacting factors.

Our study also identified a significant association between age at diagnosis and specific ADI-R scores, an “indirect” assessment measure based on information provided by the parent. Notably, we identified a limited number of previous studies that have conducted correlational analyses between ADI-R scores and age at diagnosis.

Conversely, our analysis revealed a positive correlation between the dimension of “qualitative abnormalities in communication” (scale B) and age at diagnosis, suggesting that more pronounced communication impairments are associated with a later diagnosis of autism.

The social and communication domains of the ADI-R demonstrated significant predictive capacity regarding the recognition of autistic traits (54). However, research by Hus and Lord (55) indicates that ADI-R scores can be influenced by various child characteristics; specifically, children with greater language difficulties tend to achieve higher scores. This suggests that severe language impairments may hinder families’ ability to detect early communication atypicalities, contributing to diagnostic delays, consistent with our findings. Conversely, we observed a negative correlation between “developmental anomalies evident at or before 36 months” (Scale D) and age at diagnosis. This scale includes items related to the presence of a delay in the acquisition of early words and phrases, as well as when the anomalies first became noticeable to parents and the age at which the initial evaluation was conducted. This result can be interpreted in light of the existing literature, which suggests that early recognition by the family playing a crucial role in ensuring a timely evaluation (9, 19, 27).

Consistent with existing literature, we found that early diagnosis might be hindered by the presence of comorbidities, with significant positive associations observed between the age of first diagnosis and parent-reported emotional and behavioral problems across various domains. Autistic children may exhibit associated challenges in emotional and behavioral regulation (56, 57). Numerous studies highlight elevated levels of behavioral issues (37) such as inattention, hyperactivity/impulsivity in children and adolescents with autism (58). Specifically, the externalizing manifestations of ADHD, which frequently co-occur with autism, tend to overlap with and obscure the behavioral manifestations of autistic core features; this overlap is often linked to delays in autism diagnosis (23, 24). It is important to distinguish between overlapping symptoms—commonly observed across multiple neurodevelopmental conditions, such as inattention, hyperactivity, or emotional dysregulation—and diagnostic masking, in which these co-occurring symptoms dominate the clinical presentation to the extent that core autistic traits may be misinterpreted or overlooked. In such cases, clinicians may prioritize the more overt emotional or behavioral difficulties, thereby delaying the recognition and formal identification of autism.

However, the existing literature still provides limited studies offering a clear and comprehensive definition of the emotional and behavioral characteristics that define the clinical profile of preschool-aged autistic children, with a specific focus on the correlation between these characteristics and the timing of diagnosis. In our study, we found positive correlations between the age of first diagnosis and externalizing problems such as “emotional reactivity” and “aggressive issues,” as well as internalizing problems such as those measured by the “anxiety-depression scale” and “somatic complaints,” alongside attention difficulties. Our findings diverge from those of some studies, such as that by Mandy et al. (59) who found that higher levels of ADHD-type features at age 5, along with greater emotional, behavioral, and peer relationship difficulties, were associated with early diagnosis. Although some studies have reported findings opposite to ours, prospective investigations (59) have highlighted that children with a late diagnosis, despite initially exhibiting lower levels of emotional and behavioral difficulties compared to those diagnosed early, still presented significant clinical needs that could have benefited from earlier identification. Furthermore, these studies note that the progression of emotional and behavioral difficulties varies depending on the timing of diagnosis, with a more pronounced escalation over time among those diagnosed late, who eventually develop higher levels of emotional, behavioral, and relational problems. Similar findings emerge from a large Korean cohort study (60) which reported an increased risk of psychiatric disorders in children diagnosed with autism later in life.

In light of these data, our study suggests that children with emotional and behavioral difficulties associated with core autistic traits face a higher likelihood of less favorable outcomes, due not only to the complexity of their clinical profile but also to the negative effects of delayed diagnosis. Diagnostic delays can hinder timely and targeted interventions, thereby exacerbating clinical manifestations.

The presence of unmet clinical needs linked to late diagnosis underscores the importance of further research to clarify the extent to which early diagnosis may have a protective effect against psychopathology. These findings also underscore the importance of developing clinical tools and practices capable of identifying autism even in the presence of complex emotional and behavioral profiles. Increased awareness among clinicians of how co-occurring symptoms may mask or mimic core autistic traits is essential to avoid misdiagnosis or delayed recognition. Early and accurate identification of autism, particularly in children presenting with behavioral difficulties, may enable more tailored and timely intervention strategies. Such interventions could mitigate the long-term impact of emotional and behavioral challenges and improve developmental trajectories. Furthermore, our results point to the need for systematic screening protocols in early childhood settings that take into account the broader spectrum of behavioral manifestations in autism.

In our study, we found no differences between males and females in the age of first diagnosis. However, the literature indicates that, despite the reduction of the diagnostic gap between genders over time (17), diagnoses tend to be made later in females. This delay can be attributed to several factors, including a limited understanding of autistic features in females (61), a less overt presentation of autistics features in females (16, 62), and a lower level of awareness regarding the condition among healthcare professionals (16). The unequal sex distribution in our sample, with males overrepresented, aligns with official prevalence estimates indicating a male-to-female ratio of approximately 3:1 (63). This composition may have limited our ability to detect associations between sex and age at diagnosis. Moreover, the male overrepresentation likely reflects both prevalence trends and the more subtle presentation of autistic traits in females, which complicates early identification.

While literature does not conclusively establish a distinct female autistic phenotype, some studies suggest that girls may exhibit fewer restricted and repetitive behaviors (RRBs) and comparatively stronger social and communication skills during early development, making early identification more challenging (64).

There is also growing evidence that current diagnostic criteria and screening tools may be less sensitive to female presentations of autism, which often diverge from traditional, male-based diagnostic models (64). As a result, some girls may not reach clinical assessment in early childhood, contributing to diagnostic delays and potential underrepresentation in research samples. These patterns may reflect a more complex and subtle presentation of autistic traits in females, which can hinder early detection and reduce access to diagnostic services during the preschool years.

Furthermore, literature (64) also highlights that certain sex/gender differences in RRBs may depend on cognitive functioning. For example, girls without intellectual disability tend to show fewer stereotyped behaviors than boys, whereas girls with lower nonverbal IQs may exhibit more motor stereotypies. Since our study did not stratify by cognitive functioning, future research is needed to explore how intellectual profiles may interact with sex to influence the manifestation of autistic behaviors.

These considerations underscore the need for further studies aimed at understanding sex-related diagnostic variability in early childhood.

Our sample, composed of children previously screened for indicators of autistic traits, likely reflects this male predominance, which aligns with prevalence data. It may also stem from the more challenging recognition of female autistic features, leading to under-identification and reduced access to services in early childhood.

### 4.2 Sociodemographic variables, maternal stress and age at first diagnosis

In relation to the measured sociodemographic variables, we identified a significant negative association with birth order. The literature primarily investigated the association between having an older autistic sibling and the timing of the autism diagnosis, yielding mixed results (18, 65). However, to the best of our knowledge, no studies specifically examined the relationship between birth order and autism diagnosis.

According to our findings, birth order appears to play a significant role, as firstborn children tend to receive a later diagnosis. One possible explanation is that second-born children probably benefit from their parents’ greater experience. This may include both increased familiarity with typical developmental milestones and the possibility of more immediate behavioral comparisons with an older typically developing sibling. Studies (66) indicate that later-born children, particularly second-borns, tend to exhibit more severe autistic symptomatology and greater developmental difficulties. This evidence may further explain the enhanced ability of parents to recognize symptoms earlier in later-born children, supporting the correlation observed in our study.

We did not identify any associations between the age of first autism diagnosis and other sociodemographic variables measured, including maternal and paternal age, maternal and paternal education, or number of siblings. Numerous studies have demonstrated that parental education level plays a crucial role: parents with higher levels of education are generally better equipped to navigate diagnostic services, leading to more timely diagnoses (9, 16, 17).

The absence of significant correlations between family sociodemographic characteristics and the age at first autism diagnosis may, in part, reflect the internal imbalance observed in our sample: while maternal education levels were generally high, a considerable proportion of mothers (38.62%) were unemployed; conversely, fathers showed lower average educational attainment but were almost universally employed (99.33%). These contrasting trends suggest that the indicators available in our dataset may not adequately represent the families’ actual socioeconomic status—an important limitation of the study.

Alternatively, the lack of association might be explained by a relative uniformity in access to autism diagnostic services across Italy, particularly in urban areas and specialized centers. This interpretation is supported by recent findings from Scattoni et al. (67), which highlight a relatively consistent distribution of diagnostic resources nationwide. Such structural uniformity may attenuate the influence of sociodemographic variability on diagnostic timing, thereby contributing to the non-significant associations observed in our data.

The effectiveness of local services and the quality of information provided to families—through educators, teachers, pediatricians, and specialists—may also play a role in facilitating early identification by offering warning signs and preliminary screenings. Furthermore, factors known to influence diagnosis timing (3, 9, 16, 17), such as ethnicity, geographic location, and socioeconomic status, were not accounted for in our study, which could further explain the absence of observed relationships.

Finally, our results indicate a significant relationship between the age of the first autism diagnosis and perceived maternal stress. Specifically, we observed that higher levels of parental distress (PD), which reflect the discomfort parents experience due to factors related to their parental role, as well as stress arising from difficult child behaviors (DC)—that is, the extent to which certain characteristics of the child’s behavior make them easy or difficult to manage—and overall perceived stress, were associated with a later diagnosis. This aligns with existing literature indicating that delays in obtaining an autism diagnosis are a critical factor contributing to elevated stress levels among parents, which are often higher than those experienced by parents of typically developing children or those with other chronic conditions (28, 29).

In light of all the variables considered and the correlations observed, it is likely that a single clinical variable alone is insufficient to account for the timing of diagnosis; rather, it is plausible that the interplay among clinical variables contributes to the overall expression of the autistic presentation, along with the interaction of these variables with sociodemographic and cultural factors. For example, Chen et al. (2023) (19) note that delays in cognitive and adaptive development are often linked to later diagnoses among autistic children whose mothers had low educational attainment or low family income. Consequently, the impact of cognitive or adaptive development on the age of diagnosis was moderated by the family’s socioeconomic status and the mother’s level of education.

These findings underscore the necessity of adopting a multifactorial approach to fully understand the complexities associated with autism diagnosis.

### 4.3 Predictors of age at first diagnosis

To assess whether clinical and sociodemographic factors associated with the timing of an autism diagnosis could predict an early or late diagnosis in our sample, we conducted a regression analysis including only the variables that showed significant correlations with the age at diagnosis. The analysis revealed that only two of these variables had significant predictive value. In particular, we found that IQ/DQ emerged as a significant predictor of the age at diagnosis, confirming that lower IQ/DQ scores are associated with earlier diagnoses. The timing of clinical manifestation onset and its initial recognition by the family (ADI-R, domain D) also proved to be a significant predictor, with earlier onset of autistic features associated with earlier diagnoses.

Preliminary evidence from previous studies (68) suggests that, standardized diagnostic instruments (e.g., ADOS-2) may tend to over-identify individuals with lower IQ, while concurrently under-identifying those with higher IQ.

Nonetheless, our findings align with a substantial body of literature, including a large-scale prospective study by Denisova et al. (10), which demonstrated that low IQ (below 2 standard deviations) in early infancy is a robust early indicator for children later diagnosed with autism, increasing the likelihood of diagnosis during childhood by approximately 40%. Notably, Denisova et al. also observed that children diagnosed early exhibit developmental delays as early as six months of age, unlike those diagnosed later, suggesting that early-identified cases may represent distinct phenotypic subgroups with differing neurobiological characteristics within the autism population.

Further insights from Denisova et al. (69) expand on these findings, emphasizing that children diagnosed very early not only show markedly low IQ at a young age, but also exhibit distinct neurodevelopmental features. These include significant motor delays, a higher prevalence of autism in first-degree relatives, increased rates of *de novo* mutations in genes associated with early brain development, enlarged brain volume, and cognitive challenges. The study also reinforces the importance of distinguishing early low IQ from cognitive patterns emerging in later childhood, highlighting its specific profile of global developmental impairment in both verbal and non-verbal domains with onset before age two.

Although our results do not definitively resolve this issue, they highlight that cognitive difficulties may either be linked to an earlier emergence of core autism symptoms or simply be more readily recognized by caregivers compared to more specific autistic features. This is further supported by our observation that the ADI-R domain D significantly predicts early diagnosis.

Our findings are consistent with those highlighted in a study by Harrop et al. (26), which investigated sex differences in caregiver-reported developmental milestones (first word, phrase, walking) and their contribution to the timing of initial concerns raised by caregivers and the age of diagnosis. In their study, the strongest predictor of the age of diagnosis was the age at which initial concerns were raised. The authors found that IQ was the most significant predictor of the timing of initial concerns and subsequent diagnosis, suggesting that children with lower IQ, regardless of sex, are identified and diagnosed earlier. Several studies (70) have highlighted the critical importance of parental concerns, particularly during the first year of life. These concerns, often linked to delays in achieving age-appropriate developmental milestones in language, motor skills, and social interaction, have been identified as an independent risk factor for autism. This underscores the value of regular and systematic monitoring of parent-reported concerns as a potentially invaluable component of early autism screening programs.

Together, these findings underscore how early parental recognition—particularly of cognitive delays—plays a key role in facilitating earlier autism diagnosis, while highlighting the relative invisibility of core autistic traits in early stages.

In contrast, our analyses confirmed that greater impairment in adaptive social functioning predicts a later diagnosis. To the best of our knowledge, a limited number of recent studies have investigated the predictive value of specific dimensions of adaptive functioning in relation to the age of diagnosis. Our findings suggest that difficulties in social adaptive functioning may contribute to delays in the identification of autistic features, possibly due to the nonspecific nature of the child’s adaptive difficulties, which hinder the recognition of autism-specific characteristics.

An additional hypothesis—one that warrants further investigation due to its clinical relevance—is that delayed diagnosis may further compromise the development of social competence, thereby exacerbating pre-existing adaptive difficulties over time. The absence of timely and targeted intervention may lead to a cumulative effect of unsuccessful social experiences, progressively reducing opportunities for social learning and negatively influencing the developmental trajectory of interpersonal functioning.

Conversely, other clinical and sociodemographic factors correlated with age at diagnosis did not significantly predict diagnosis timing. Among clinical factors, these include qualitative communication variations (ADI-R, domain B) and emotional-behavioral aspects such as emotional reactivity, anxious/depressed symptoms, somatic complaints, attention problems, and aggressive behaviors. Sociodemographic variables, including birth order, also showed no significant predictive value. Although these characteristics may correlate with diagnosis age, they do not reliably distinguish between early and late diagnosis.

Overall, these results emphasize the multifactorial nature of autism diagnosis timing and the importance of integrating multiple clinical dimensions to improve early identification.

## 5 Limitations and future research

### 5.1 Sample representativeness and generalizability

The relatively small sample size, the retrospective design, and the recruitment from a single geographic area may limit the generalizability and representativeness of our findings. Additionally, individuals who tend to be diagnosed later—such as females or those with higher adaptive functioning—are likely underrepresented in early childhood samples like ours. This may influence observed patterns in age at diagnosis and should be considered when interpreting the results. Although the sex distribution in our sample reflects official prevalence estimates, the overrepresentation of males may have reduced our ability to detect potential sex-related differences in diagnostic timing.

### 5.2 Missing variables

Another limitation is the lack of data on language development. Given its relevance to the developmental trajectories of children with an autistic condition, examining its relationship with age at diagnosis could provide valuable insights into early identification processes. Future research should address this gap to enhance our understanding of diagnostic timing.

Additionally, the absence of comprehensive socioeconomic data limits our ability to assess the role of broader contextual and structural factors influencing the timing of diagnosis. While we collected and analyzed some sociodemographic variables such as parental age, educational level, and employment status—which are often used as approximate proxies for socioeconomic status (SES)—we did not directly assess SES through these indicators. More specific SES-related factors, including family income, geographic disparities, and access to healthcare services, were not included in our study. This limitation constrains our capacity to fully explore the complex socioeconomic influences on diagnostic timing and should be addressed in future research.

Moreover, due to the retrospective nature of the study, it was not possible to determine whether the initial pediatric referral was prompted directly by parental concerns regarding autism-specific signs. Although all children were referred following pediatric suspicion, we could not systematically assess whether parents themselves recognized early autistic features. This limitation restricts our understanding of the influence of parental awareness on the diagnostic process. Future prospective studies should investigate this aspect using structured interviews or validated questionnaires.

### 5.3 Measurement limitations

Due to the retrospective nature of the study, it was not possible to access the ADI-R assessment for all participants, as this specific tool was not consistently administered or documented in clinical records. This represents a limitation in the uniformity and completeness of diagnostic data.

Furthermore, the cross-sectional nature of the dataset limits conclusions about developmental trajectories, highlighting the need for longitudinal research to better understand changes over time.

### 5.4 Future directions

Future research should aim to expand the sample size and include participants from multiple centers across different geographic areas of Italy. This would enhance representativeness and enable broader investigation of sociodemographic factors influencing age at diagnosis.

Longitudinal studies will also be crucial in evaluating the persistence and long-term impact of the individual and contextual variables explored in this study.

Our findings underscore the importance of improving early detection efforts, particularly for groups less likely to receive timely diagnosis. This includes developing screening instruments better tailored to identify diverse autistic presentations in early childhood, which could significantly enhance access to early intervention.

## 6 Conclusion

This study confirms that the timing of autism diagnosis remains a complex challenge influenced by multiple clinical and sociodemographic factors, some of which show significant associations with early or late diagnosis, although only a few possess true predictive power.

Cognitive level, measured by IQ/DQ, emerged as the most robust predictor of age at diagnosis, with lower cognitive functioning associated with earlier diagnosis. This likely reflects the greater visibility of global developmental delays, facilitating timely recognition by both families and healthcare professionals. Similarly, the early onset of autistic features and their initial recognition by caregivers (measured by ADI-D) were significant predictors, underscoring the critical role of caregiver knowledge, awareness, and attentive early observation in enabling prompt access to diagnostic pathways.

Some clinical variables, although lacking predictive power, are associated with delayed diagnosis. These include the characteristic emotional-behavioral profile of autism—such as reactive emotionality, anxiety, aggressive behaviors, and attentional difficulties—and lower levels of social adaptive functioning. These factors may impede early detection due to their nonspecific nature and overlap with core autistic symptoms.

Among the sociodemographic factors examined, birth order emerged as a relevant variable: second-born children were more likely to receive an earlier diagnosis, reaffirming the central role of familial experience and observational sensitivity. Additionally, higher levels of maternal stress were significantly associated with later diagnoses, highlighting the psychological burden that diagnostic delays can impose on families. Within the Italian context, no significant associations were found between age at diagnosis and other sociodemographic variables such as parental age, level of education, or number of siblings.

It is important to note, however, that although birth order and maternal stress showed statistically significant associations with the timing of diagnosis, neither demonstrated true predictive power within our model. With appropriate interpretative caution, it may be hypothesized that these associated variables interact in complex ways with other clinical and sociodemographic factors, indirectly influencing the diagnostic timeline. Further investigations are warranted to better understand the nature and direction of these potential interactions.

It is important to note that in children with average or mildly reduced cognitive functioning, autistic traits may be less overt and remain unrecognized during the preschool years, when environmental demands have not yet exceeded the child’s capacities. Nonetheless, this period remains crucial for timely intervention aimed at preventing adverse outcomes.

These findings emphasize the need for a multifactorial and integrated approach to understanding the determinants of diagnostic timing. Enhancing clinical screening practices with tools capable of detecting subtle communicative differences and considering the qualitative variability of autistic phenotypes could help reduce diagnostic delays and improve long-term functional outcomes for autistic children and their families.

## Data Availability

The raw data supporting the conclusions of this article will be made available by the authors, without undue reservation.
